# Scientometric Analysis and Combined Density-Equalizing Mapping of Environmental Tobacco Smoke (ETS) Research

**DOI:** 10.1371/journal.pone.0011254

**Published:** 2010-06-22

**Authors:** Karin Vitzthum, Cristian Scutaru, Lindy Musial-Bright, David Quarcoo, Tobias Welte, Michael Spallek, Beatrix Groneberg-Kloft

**Affiliations:** 1 Department of Information Science, Institute of Occupational Medicine, Charité-University Medicine Berlin, Free University Berlin and Humboldt-University Berlin, Berlin, Germany; 2 Department of Respiratory Medicine, Hanover Medical School, Hanover, Germany; 3 Otto-Heubner-Centre, Charité-Universitätsmedizin Berlin, Free University Berlin and Humboldt-University Berlin, Berlin, Germany; Chiba University Center for Forensic Mental Health, Japan

## Abstract

**Background:**

Passive exposure to environmental tobacco smoke (ETS) is estimated to exert a major burden of disease. Currently, numerous countries have taken legal actions to protect the population against ETS. Numerous studies have been conducted in this field. Therefore, scientometric methods should be used to analyze the accumulated data since there is no such approach available so far.

**Methods and Results:**

A combination of scientometric methods and novel visualizing procedures were used, including density-equalizing mapping and radar charting techniques. 6,580 ETS-related studies published between 1900 and 2008 were identified in the ISI database. Using different scientometric approaches, a continuous increase of both quantitative and qualitative parameters was found. The combination with density-equalizing calculations demonstrated a leading position of the United States (2,959 items published) in terms of quantitative research activities. Charting techniques demonstrated that there are numerous bi- and multilateral networks between different countries and institutions in this field. Again, a leading position of American institutions was found.

**Conclusions:**

This is the first comprehensive scientometric analysis of data on global scientific activities in the field of environmental tobacco smoke research. The present findings can be used as a benchmark for funding allocation processes.

## Introduction

Among other occupational [1], [2], [3] and environmental [4], [5], [6] noxious substances, ETS is known as a major airborne health hazard.

The severe short- and long-term impact of ETS on the onset and progression of life-threatening diseases has been the focus of numerous studies and publications.

Passive smoking is defined as the involuntary inhalation of tobacco smoke. It is also called secondhand smoke, sidestream smoke or environmental tobacco smoke (ETS) and has been shown to exert a major burden of disease, especially as concerns respiratory diseases [5], [7]. The likelihood of exposure to ETS varies according to socio-demographic groups, individual health behaviors, and the type of smoking restrictions in workplace.

Current legal regulations like the ban of smoking in restaurants and the workplace may lead to a decline in epidemiologic data in future. However, so far the toxic effect and mode of action of ETS has not been fully investigated or understood. Long-term effects will remain and will still require treatment and scientific observation, especially considering rising numbers of smokers and less restrictive laws in Asian countries, like China [8], [9]. Therefore, research in the area of environmental tobacco smoke is still urgently needed.

Although many governments' reaction to this health hazard has been slow or inadequate, research in this area has already been intensive over the years. Numerous reviews have focused on single aspects of ETS. Especially the protection of children and infants (e.g. after an intrauterine exposure), who are at a higher risk than adults of being affected by ETS, has been a target of modern health policies. These range from dangers posed to unborn children, their later social behavior and success in school, the impact on diseases such as diabetes, coronary heart diseases, as well as the onset or progression of strokes and cancer [10], [11], [12], [13], [14], [15], [16], [17]; Consequences of ETS on cognitive impairment and related diseases, such as Alzheimer's and Parkinson's disease, are presently under investigation [17], [18], [19].

In 2004, a study by the International Agency for Research on Cancer of the World Health Organization reported that nonsmokers are exposed to the same carcinogenic compounds as smokers. Environmental tobacco smoke contains more than 4,000 chemicals, of which 69 are known carcinogens such as formaldehyde, lead, arsenic, benzene, and radioactive polonium-210. In fact, according to research by tobacco companies, some of these known carcinogens have been shown to be present at higher concentrations in secondhand smoke than in firsthand smoke [17], [20], [21].

To measure ETS exposure quantitatively, serum and urine concentrations of cotinine and amount of time exposed to ETS (at home and in the workplace) were analyzed [16], [22].

Despite growing interest and research output in this field, the scientific data that has been published to date on ETS has not been dissected in detail by means of scientometrics. By contrast, the existing scientometric studies have had a more general focus [23], [24]. Scientometrics is defined as the measurement and analysis of science, often using bibliometrics, the measurement scientific publications. Modern scientometrics are based on studies by de Solla Price and Garfield [25], [26]. The latter also founded the Institute for Scientific Information with the database Web of Science [27], [28]. It was demonstrated that scientometrics, which includes use of mathematical techniques to investigate publishing and communication patterns in the distribution of information, has been an established approach in occupational and industrial health for about 20 years [29].

Since there is no in-depth scientometric analysis on ETS at present, the objective of the current study was to analyze scientometric parameters in the field of ETS research. Specifically, the study assessed the following qualitative and quantitative measurements: a) Total number of published items and citations, b) average authorship numbers, c) countries' total numbers of published items, d) countries' average citation indices, e) research networks per country f) institutional networks and g) journals and papers specifications.

## Results

### Total numbers of articles, citations, average citation rates and average authorships

**Figure 1:**
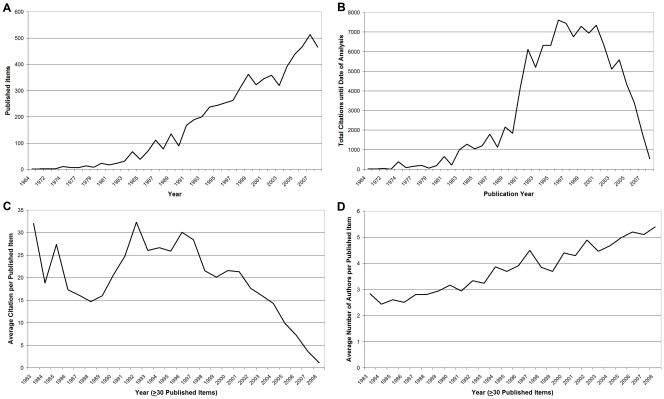
Article related bibliometric data. (A) shows the analysis of total number of published items (B) shows the analysis of total number of citations (C) shows the annual average number of citations per article. (D) shows the average number of authors per article per year.

The total number of publications is an indicator of quantitative research productivity. A total of 6,580 “environmental tobacco smoke”-related articles were identified in the Web of Science database. The first article was published in 1964. To date, the most articles published within a given year were published in 2007 (513), followed by 2006 (468) and 2008 (465) (Figure 1a). In terms of total numbers of citations, articles published in the year 1996 take the lead with 7,614 citations followed by citations in the years 1997 (7439), 2001 (7354) and 1999 (7285) (Figure 1b). When analyzing the average number of citations per published item per year since 1983, values ranged between 1.15 citations per article in the year 2008 and 32.32 in the year 1992 (Figure 1c). Concerning the average number of authors per article between 1983 and 2008, a range was found between 2.44 in the year 1984 and 5.4 authors per article in the year 2008 (Figure 1d).

### Research analysis by country

**Figure 2:**
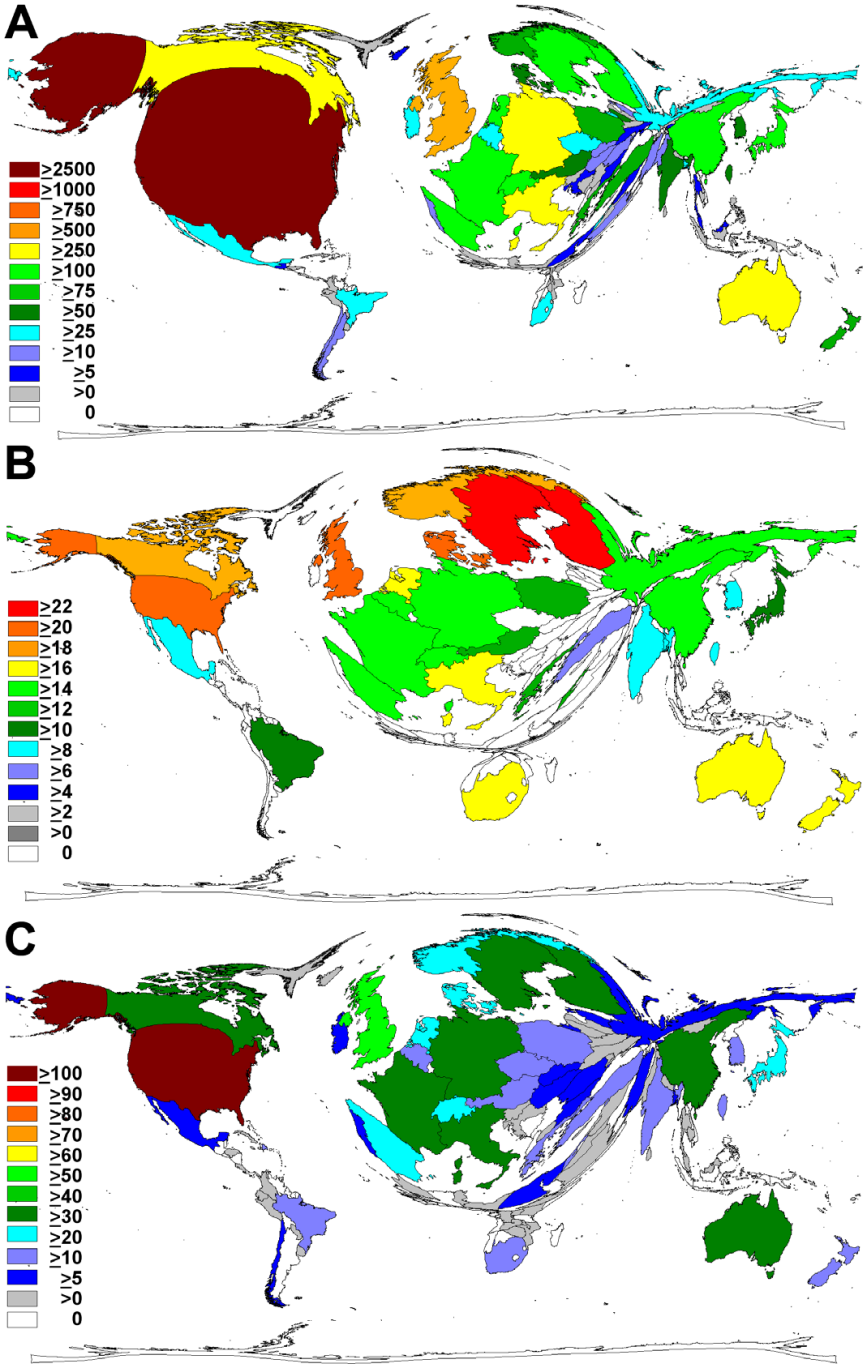
Country specific analysis. All maps show scales proportionately to the specific parameter and country. Colors mark total number of contributions (A), the average citations per article (B), and the countries' h-indices (C). (A) Map is illustrating the number of articles pertaining to environmental tobacco smoke published per country from 1900 to 2008. (B) Map is illustrating the countries' average citation rates per publication for environmental tobacco smoke-related articles from 1900 to 2008. (C) Map is illustrating the country-modified h-indices from 1900 to 2008.

The United States of America is the highest-ranking country in terms of overall publications, with a total of 2,959 articles. The UK is in second place with 629 articles, followed by Germany (396), Canada (289), Australia (263) and Italy (262). Using density-equalizing mapping, countries were resized proportional to the total number of publications to illustrate their research output (Figure 2a). In this visualization, the USA clearly dominates the cartogram.

Similar results were found for the analysis of countries' average citations per ETS-related article. Articles originating from Sweden and Finland had the highest average citation rate, with 22.91 and 22 average citations, respectively, followed by American articles with 21.29, and Danish articles with 21.20 and articles from the UK with 20.42. These results are depicted by density-equalizing mapping in Figure 2b, which differs from the cartogram for total number of published items in Figure 2a.

In the analysis of the country-modified h-indices, the United States ranked highest with an h-index of 109, followed by the UK with 54, and Sweden and Germany, each with 39. These results are also presented in form of a cartogram (Figure 2c).

### Country research network analysis

**Figure 3:**
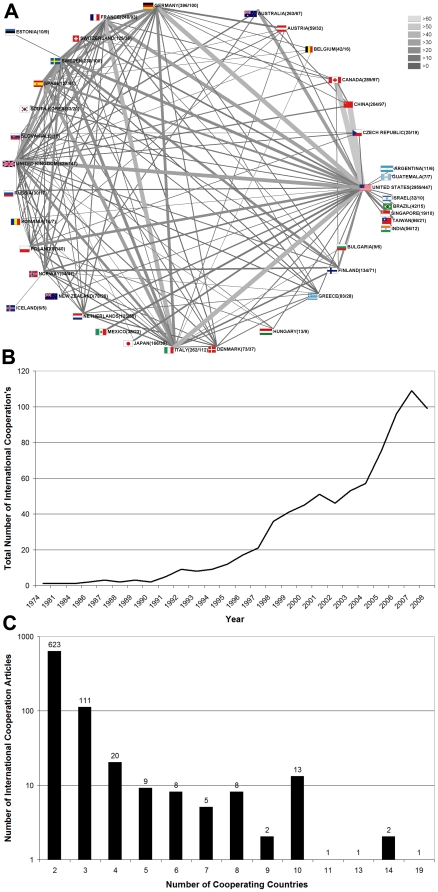
Country network analysis. (A) Radar chart depicting bilateral networking between countries for the overall numbers of cooperation between the two countries. Grayscale shade and width of the connecting lines represent the number of bilateral cooperative research efforts. (B) Evolution of international cooperation over the years since 1974. (C) Total numbers of published items with authors originating from two, three or more countries (bi-, tri-, and multilateral cooperation).

There is an overall noticeable increase in international research cooperation. In 1974, the first ETS-related article resulting from international cooperation was published. 2007 represents the year with the largest number of cooperation articles (109), followed by 2008 (99) and 2006 (96) (Figure 3b). To visualize research networks for “environmental tobacco smoke” articles, the radar charting technique was used. With a total of 57 articles resulting from bilateral cooperation, Canada and the United States and China and the United States were found to be the leading cooperative partners. They were followed by the cooperation between Italy and the United States (45), the United States and France (45) and Germany and the United States (44) (Figure 3a). The majority of these articles are the result of bilateral cooperation (623). One hundred and eleven articles are the result of a trilateral collaboration and twenty articles originate from the collaboration between four countries (Figure 3c).

### Quality of international research networks

**Figure 4:**
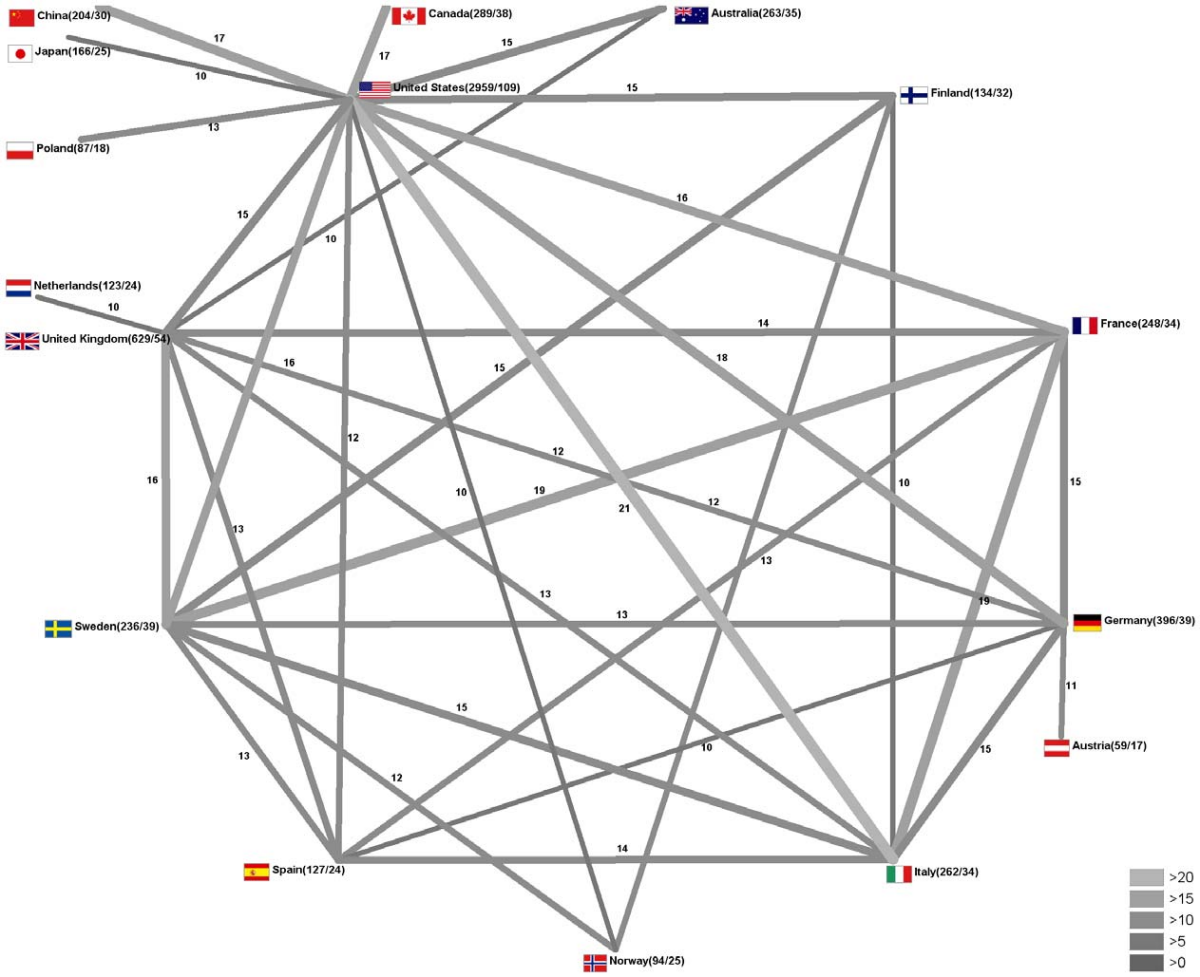
Qualitative analysis of countries' research networks. Radar chart visualization of the h-indices of bi- and multilateral cooperation between different countries. For the sake of clarity, values under 10 are not depicted. Numbers following country name represent the total number of published items/country-related h-index.

Cooperation between the USA and Italy had the highest h-index value (21). Cooperation between France and Sweden and France with Italy, each had h-indices of 19. The h-index of cooperation between Germany and USA was 18, followed by Canada and USA and China and USA, each with 17 (Figure 4).

### Institutional research network analysis

**Figure 5:**
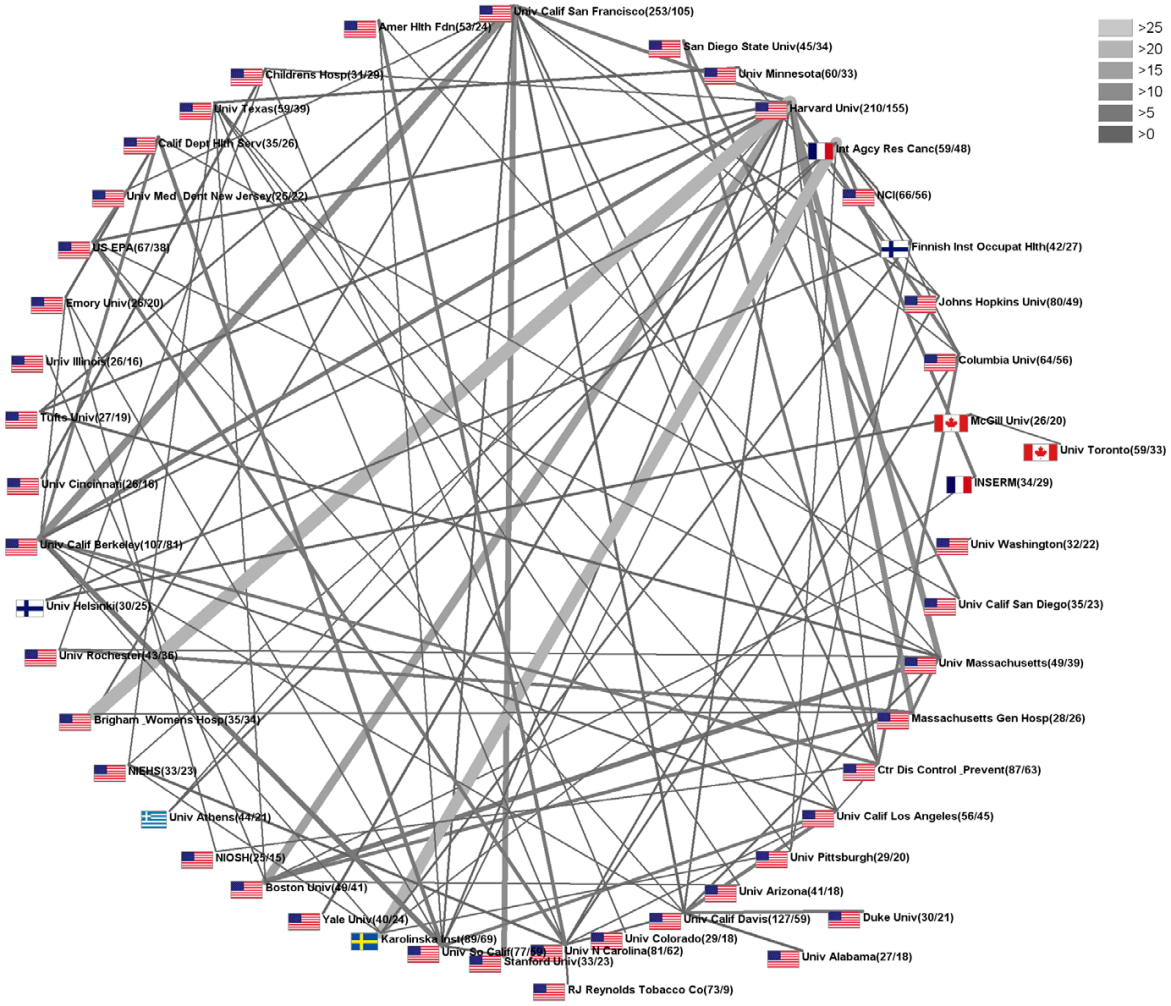
Institutional research network analysis. Radar chart visualization of cooperation between different institutions. Grayscale shade and width of connecting lines depict the number of cooperative research efforts. Cooperation resulting in fewer than three articles was not depicted.

The radar charting technique was also used in order to identify and depict the leading institutional networks (Figure 5). The highest numbers of cooperation between institutions were present for cooperative research efforts between the Harvard University and Brigham Women's Hospital, resulting in 25 ETS-related articles. In second place, 22 articles resulted from the cooperation between International Agency for Research on Cancer and Karolinska Institute. The third most productive cooperation was between the Massachusetts-based Boston and Harvard Universities with 16 published items.

### Average length of ETS-related articles

Only years with at least 30 published items were computed in order to filter out distortion due to outliers. Since 1983, length of articles has increased almost steadily from roughly five to nine pages in 2008 (Figure 5). In 1986 and in 1998 minor troughs are observable. The nine-page peak was reached for the first time in 2000.

### Journal analysis

**Figure 6:**
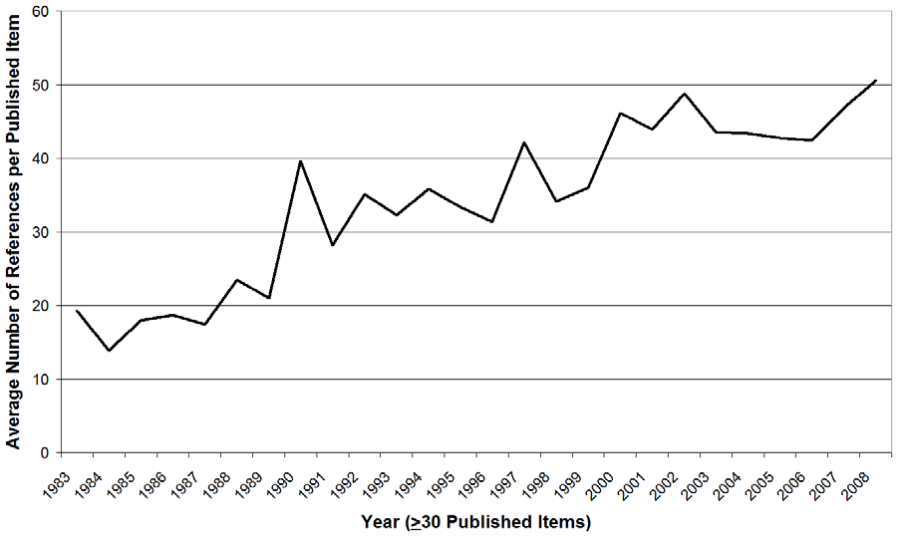
Average length of articles. Average number of pages of all in this analysis included articles since 1983 are shown in the diagram below.

**Figure 7:**
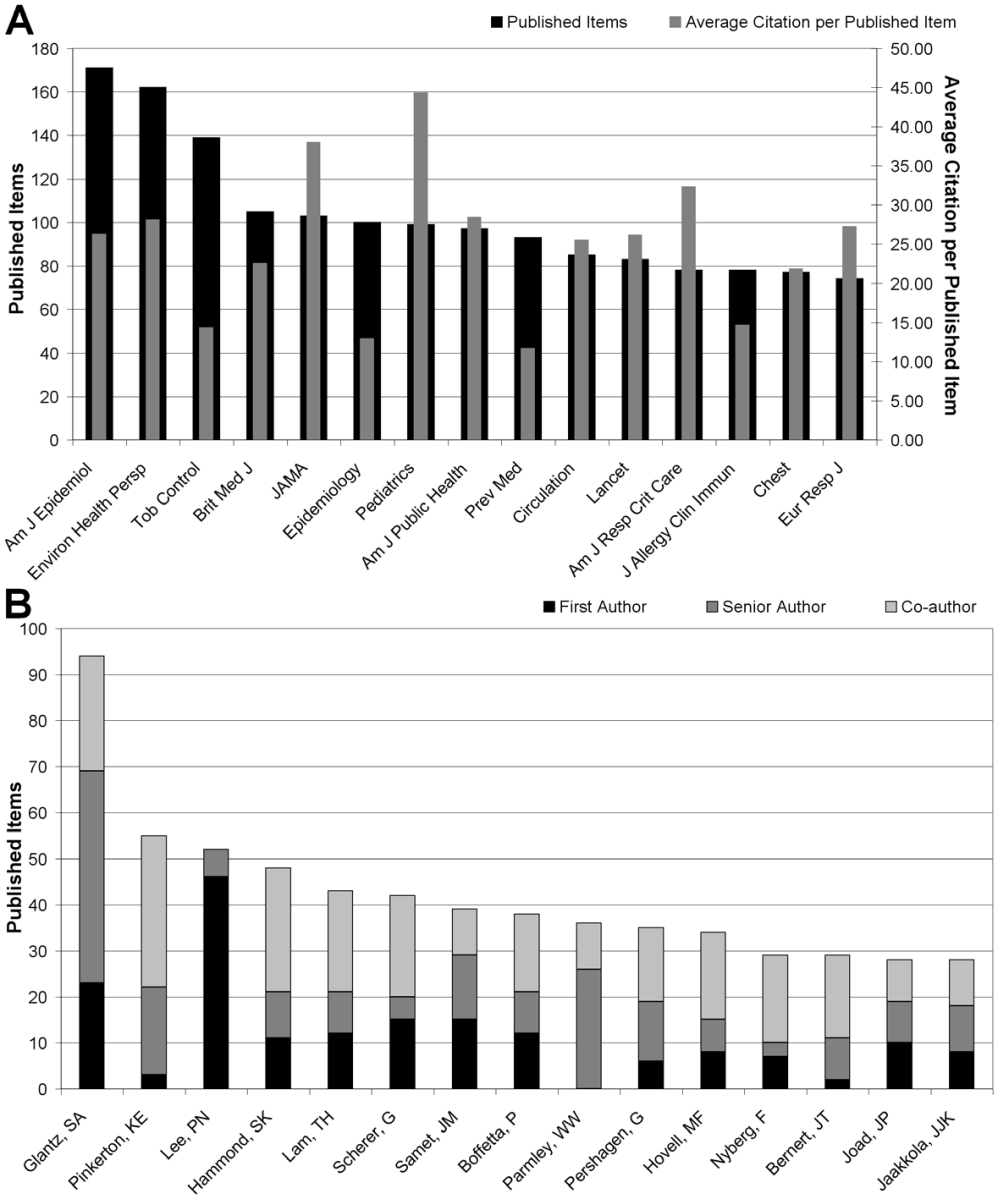
Top 15 journals and authors analysis. (A): Ranking of most productive journals. (B): Ranking of most productive authors regarding their position on author's list.

The most ETS-related articles were published in the American Journal of Epidemiology (171), followed by the “Environmental Health Perspective” (162), “Tobacco Control” (139), “British Medical Journal” (105) and “Journal of the American Medical Association” (103) (Fig. 6a). The “Pediatrics” journal holds the highest rate of 44.29 citations per ETS-item published, followed by the “Journal of the American Medical Association” (37.98) and “American Journal of Respiratory Critical Care” (32.32) (Figure 7).

### Author analysis

In the final step of the scientometric ETS-assessment, an analysis was performed for all the authors of “environmental tobacco smoke”-related articles. The researcher Glantz, SA was found to author or co-author the highest number of articles related to “environmental tobacco smoke” (94), followed by Pinkerton, KE (55), Lee, PN (52) and Hammond, SK (48) (Figure 6b). Glantz, SA also had the highest number of senior authorships (46), followed by Parmley, WW (26) and Pinkerton, KE (19). Lee, PN wrote the highest number of articles as first author (46), followed by Glantz, SA (23) (Figure 7b).

## Discussion

Among other occupational [1], [2], [3] and environmental [4], [5], [6] noxious substances, ETS is known as a major airborne health hazard.

The severe short- and long-term impact of ETS on the onset and progression of life-threatening diseases has been the focus of numerous studies and publications. Especially the protection of children and infants (e.g. after an intrauterine exposure), who are at a higher risk than adults of being affected by ETS, has been a target of modern health policies. Current legal regulations like the ban of smoking in restaurants and the workplace may lead to a decline in epidemiologic data in future. Further steps in banning smoking from private cars, public beaches and places, even a restriction for logo branded cigarette packets are already in current public debate in the western world [30], [31], [32]. However, so far the toxic effect and mode of action of ETS has not been fully investigated or understood. Long-term effects will remain and will still require treatment and scientific observation, especially considering rising numbers of smokers and less restrictive laws in Asian countries, like China [8], [9]. Therefore, research in the area of environmental tobacco smoke is still urgently needed. To compare scientometric results we composed a table showing other relevant data on smoking behavior and economic facts for leading scientific countries (table 1) to highlight the discrepancy in research and actual smoking prevalence [33], [34].

**Table 1:** This table shows data of Gross domestic income in 2008, population estimates of 2006 and smoking prevalence in 2004 for leading scientific countries in ETS research.

Country or area	Per Capita GDP (US$) 2008	Population estimates 2006 (in thousands)	percentage of male smoker/2004	percentage of female smokers/2004
Australia	48.253	20.409	below 20%	10–19%
Canada	45.166	32.312	20–29	10–19%
Denmark	62.520	5.435	30–39	20–29
Finland	51.409	5.246	20–29	10–19%
Germany	44.363	82.464	30–39	20–29
Italy	38.640	58.607	30–39	10–19%
Sweden	52.035	9.030	below 20%	below 10%
United Kingdom	43.544	60.238	20–29	10–19%
United States	45.230	299.398	20–29	20–29%
China	3.292	*1.311.020*	above 60%	10–19%

Gender differences in smoking behavior are added in column 3 and 4.

While previous scientometric studies have assessed research in the field of tobacco and smoking in general [23], [24], [35], the present study focuses on the field of environmental tobacco smoke research using a combination of novel visualizing tools, such as density equalizing mapping, and classic scientometric tools, such as publication and citation analysis.

Smith et al. recently addressed the issue of scientometric research efforts in the area of environmental and occupational health [36] and has been a recommended approach in occupational and industrial health since 1990 [29]. In this respect, McCunney and Harzbecker published a study on citation patterns in 1992 [37]. New proposals for improved measures of indices for the assessment of research output were published by a number of scientists [38], Garfield [39] and Gehanno and Thirion [40].

These findings need to be interpreted in the context of ETS research and funding: passive smoke-related diseases are estimated to exert a major burden of disease, which is costly in terms of diagnosis and therapy. Therefore, a number of national and international ETS research networks were created by governmental and non-governmental institutions. These networks are responsible, at least in part, for the increasing amount of multilateral cooperation, which was found to be responsible for ETS-related publications in our analysis.

The generalizability of our ETS research analysis is limited by the use of the Web of Science database. Although it is part of the largest global biomedical databases, there are still important publications that cannot be accessed through this system, which may present a selection bias. However, due to the relative magnitude of this database, it can be hypothesized that the research trends we observed in the present study represent ongoing research efforts in this field. Nevertheless other databases like PubMed, Scopus, Google Scholar etc. should be included in further research endeavors. In general, the USA ranked highest in terms of quantitative research efforts, which was evident as depicted by density-equalizing mapping techniques and by using the Gastner and Newman's algorithm [41]. This technique also illustrates that by applying other measures of research output, such as the average citation rate per country, other countries such as Sweden and Finland take a leading position.

Whereas the number of published items was currently considered as a quantitative measure of research productivity (showing American dominance in this field), citation analysis may be used as an indicator for research quality. However, qualitative indicators need to be regarded critically and, therefore the data should not be over-interpreted, as described in numerous previous articles [38], [39], [40].

Concerning the length of ETS-related articles, we can draw no conclusions as to the causality of the observed increases in average article length, whether these increases reflect advances in research productivity or changes in journals' policies. It is therefore difficult to judge, whether new journal policies concerning article length have changed the observed publishing behavior, or vice versa.

Modern technical support, such as the accessibility of the Internet, electronic scientific databases, software like Endnote and online submission options, considerably accelerate publication procedures. New trends in publication behavior, like a tendency toward rising citation rates and longer articles, were observed within this analysis. Increasing numbers of co-authorships and self-citations are very likely an indirect result of modern scientific funding policies, encouraging researchers to adopt inflationary publishing behaviors [42], [43].

The present study represents a large-scale scientometric analysis of “environmental tobacco smoke” publications, using Web of Science database, and visualization of the results, using density-equalizing calculations and charting techniques. The objectives of this study were to analyze total numbers of ETS-related articles and citations, average number of authorships, country-specific research productivity, average citation indices, as well as international and institutional research networks and to detect researching trends over the past decades. We report strong increases in this area of research in terms of productivity and networking over the years but underlining the USA's leading role in research. Although quantitative increase is not necessarily a proof of scientific progress, but can be interpreted as a constraint in modern research funding policies as institutes with higher publication numbers in more impact-factor journals and more prolific authors will be funded more probably [44], [45]. The established techniques can be of use for future scientometric studies in this field. Our results should be related to other subject areas of interest in health prevention or synonyms of ETS to enable a more profound interpretation of ETS research trends and as a consequence to highlight a lack of potential specific research objectives. These findings should then be taken into consideration for future research project fundings.

## Materials and Methods

### Data source and time span

Data were retrieved from the Thomson Reuters' database Web of Science [27], [28]. Data analysis was restricted to articles published from 1900 to 2008; data entries from 2009 were excluded from the analysis, because acquisition for this year was not yet complete.

### Search strategies

All published items matching the English term “environmental tobacco smoke*” (in the “topic” search field) were retrieved and downloaded for analyzing purposes using a web interface (last update: 2009-05-05). No additional filters were used. Preliminary searches (1999–2008) proved ETS (3,995 items) being the most common term in use compared to secondhand smoke (353 items) or passive smoking (2,462 items) etc., which led us to this analysis.

### “Quality” parameter analysis

For countries with at least 30 published items on “environmental tobacco smoke”, the average citation rate per published article was calculated. Countries publishing less than 30 articles might distort representative results due disproportionately high citation rates.

The original h-index for authors, as defined by Hirsch [46], is as qualitative index (h), where h is the factor of articles written by a given author, if each one of these h articles has been cited at least h times. It is useful for the assessment of both productivity and quality of scientific output. This h-index was modified and extrapolated for all articles originating from a specific country. In this respect, countries' modified h-indices were calculated in order to assess the quality of articles from specific countries.

### Density-equalizing mapping

Density-equalizing mapping procedures were applied to this study, as described in previous studies [47], [48]. In brief, all territories were resized according to 1) the number of items published pertaining to “environmental tobacco smoke”, 2) the average citation rates and 3) the modified country h-index, respectively [49]. For the resizing procedure, each country's area was scaled in proportion to these variables. The calculations were based on Gastner's and Newman's algorithm [41].

### Analysis of bi- and multilateral country and institution cooperation

The analysis of bi- and multilateral international and institutional cooperation was selected to investigate ETS research networks. A bilateral cooperation was found, if at least one of the authors' country of origin for a given publication differs from that of any of the co-authors. A matrix with all countries identified was compiled with the appropriate values for the cooperation for each country pair. A second software module was developed to evaluate the matrix and transform these figures into vectors. The width of the vector corresponds the number of articles resulting from cooperation between these two countries. For the sake of clarity, the threshold for the multilateral country analysis was set at least five cooperating countries and the threshold for the analysis multilateral cooperation among institutions analysis was set at least four cooperating countries. The results were depicted graphically using the radar charting technique.

### Modified h-index for international cooperation

To assess the quality of international cooperation, the theory of the h-index by Hirsch [46] was extrapolated and a similar index was computed for each bi- and multilateral cooperation. For the articles identified in the analysis of bilateral international cooperation, the h-index was computed and saved into a matrix containing all cooperating countries. Analogue to analysis of bilateral international cooperation, the information in this matrix was graphically depicted in a radar chart.

### Analysis of length of articles

The analysis of article length was performed in order to identify quantitative differences in article length over time in the field of environmental tobacco smoke research. Diagrams were used to highlight a steady increase in article length.

### Journal and author analysis

In order to identify the journals and authors with the highest publication output in the field of environmental tobacco smoke, the set of published items was analyzed according to the publishing journal and contributing authors. The journals were also evaluated according to the numbers of citations. The authors were analyzed according to the type of authorship (first-, senior- or co-authorship).
